# Factors Associated with Exclusive Use of Hygienic Methods during Menstruation among Adolescent Girls (15–19 Years) in Urban India: Evidence from NFHS-5

**DOI:** 10.1016/j.heliyon.2024.e29731

**Published:** 2024-04-16

**Authors:** Doli Roy, Nuruzzaman Kasemi, Manik Halder, Malasree Majumder

**Affiliations:** a Department of Geography, Raiganj University, Raiganj, 733134, West Bengal, India; b Department of Political Science, Raiganj University, West Bengal, India

**Keywords:** Reproductive health, Menstrual hygiene methods, Adolescent girls, Urban India, NFHS-5

## Abstract

**Background:**

Menstrual hygiene is a critical public health concern for adolescent girls in urban India. However, there is a paucity of research on this subject, particularly on a national scale. To the best of our knowledge, this study diverges from previous research, as the majority of prior investigations in India have centered on rural locales, married individuals, and those aged between 15 and 24 years. Thus, this study aims to fill this gap by investigating the factors associated with the exclusive use of hygienic methods during menstruation among urban adolescent girls (15–19 years) in India.

**Method:**

A total of 25136 samples were included in this analysis from the National Family Health Survey 5 (NFHS-5). The Binary logistic regression model has been administered to determine the associated factors of the exclusive use of hygienic methods among adolescent girls.

**Results:**

The results of the multivariate model revealed significant positive associations between higher education levels and usage of menstrual hygiene management products (AOR: 1.860; 95 % CI: 1.418–2.439), (AOR: 2.110; 95 % CI: 1.553–2.867). Additionally, individuals in higher wealth quintiles were more likely to use MHM products, with the richest quintile showing the highest likelihood (AOR: 5.310; 95 % CI: 4.494–6.275). Attendance at cultural events such as cinema or theater was positively associated with MHM product utilization (AOR: 1.338; 95 % CI: 1.181–1.517).

Conversely, Lack of access to sanitation facilities was inversely associated with MHM product utilization (AOR: 0.742; 95 % CI: 0.628–0.877). Muslim girls had lower odds than Hindus (AOR: 0.576; 95 % CI: 0.520–0.637). Substantial regional variations were evident, with the Western (AOR: 0.879; 95 % CI: 0.759–1.019), Eastern (AOR: 0.747; 95 % CI: 0.654–0.854), Central (AOR: 0.349; 95 % CI: 0.313–0.388), and North-eastern regions (AOR: 0.597; 95 % CI: 0.490–0.727) displaying diminished odds of MHM product usage relative to the southern region. General caste had higher odds compared to scheduled caste (AOR: 1.255, 95 % CI: 1.103–1.429), while other backward caste had lower odds (AOR: 0.858, 95 % CI: 0.771–0.955).

**Conclusion:**

These findings underscore the importance of addressing inequalities in access to menstrual hygiene products among urban adolescent girls in India. Targeted interventions and educational programs are essential to ensure equitable access and promote overall health and well-being.

## 1 Introduction

Menstruation is a natural and significant physiological process that every woman experiences throughout her reproductive years and poses unique challenges for adolescent girls, particularly those aged 15 to 19 [1,2]. Firstly, inadequate access to basic sanitation facilities poses a significant obstacle to maintaining proper menstrual hygiene management. Many girls, especially from marginalized regions or socio-economic backgrounds, lack clean and private toilet facilities, complicating hygienic menstruation management [3]. Secondly, limited access to menstrual hygiene products remains a critical concern. Issues related to affordability, availability, and quality of sanitary pads or tampons may force some girls to resort to unhygienic alternatives such as old rags or leaves. Thirdly, sociocultural taboos and stigma surrounding menstruation further compound these difficulties, hindering open discussions about menstrual hygiene methods (MHM) and impeding access to information and resources [4]. Lastly, insufficient guidance and knowledge exacerbate these challenges. A lack of comprehensive education on menstruation and MHM practices can perpetuate misconceptions and improper management strategies, posing potential health risks.

One of the most concerning outcomes of these challenges is the high rate of school dropout and absenteeism among adolescent girls. Without proper menstrual hygiene management resources, girls often miss school days during their menstruation, leading to academic underachievement and, in many cases, discontinuation of education altogether [5]. In urban India, where modern amenities are more accessible, addressing these challenges is crucial. Despite improved infrastructure, adolescent girls still face barriers to maintaining proper menstrual hygiene. Therefore, exploring the factors influencing the exclusive use of hygienic products during menstruation among adolescent girls is imperative.

Globally, the availability and use of MHM among adolescent girls vary across countries and regions [[6], [7], [8]]. For instance, in Ghana, a considerable proportion of schoolgirls in the northern region resort to using reusable cloth pads (44 % and 54.2 %) due to inadequate access to menstrual hygiene facilities, such as safe and clean sanitary products [9]. A study conducted in 2015 in Kenya revealed that 65 percent of girls and women were unable to afford sufficient menstrual products, as the cost of a package of sanitary pads equaled the daily wage of an unskilled worker [10]. In Pakistan, menstrual hygiene management poses a significant challenge, especially in low-income communities, where women and girls often lack access to basic menstrual hygiene products. According to UNICEF, 44 % of girls do not have access to basic menstrual hygiene facilities at home, in their workplace, or at school [11]. In low-income countries, it is estimated that only 1 in 10 girls have access to sanitary pads [12]. In some cases, girls use unhygienic substitutes like old rags, leaves, or other unsafe materials. Over the years, there has been some progress in using MHM during menstruation among adolescent girls in India [13]. But there are still challenges that need to be addressed. According to the National Family Health Survey (NFHS-4) conducted in 2015–2016, around 58 % of urban adolescent girls in India reported using MHM during menstruation, which includes sanitary pads, tampons, or menstrual cups [14]. However, this figure varies across different regions and socio-economic backgrounds. In Indian society, people are often hesitant to talk openly about menstruation. However, it is important to recognize the significance of menstrual hygiene as a starting point for addressing broader issues such as women's empowerment and gender equality. Every country should prioritize eliminating all forms of discrimination against women, including social norms related to menstruation. It should be openly acknowledged and discussed. Adolescent girls can realize their full potential and contribute to society by breaking the silence surrounding menstruation.

There has been a growing recognition of the importance of menstrual hygiene and efforts to improve access to MHM in India. Initiatives such as the Swachh Bharat Abhiyan and the Beti Bachao, Beti Padhao campaign have aimed to raise awareness about MHM and provide access to sanitary products [15]. The Government of India also launched the Menstrual Hygiene Scheme, which provides free sanitary napkins to adolescent girls. However, despite these efforts, there are still barriers to achieving exclusive use of MHM among adolescent girls in India. An increasing body of literature has scrutinized various determinants impacting the utilization of MHM practices among adolescent girls, encompassing factors such as social group, education level, household wealth, geographical location, and media exposure [[16], [17], [18], [19], [20], [21], [22], [23], [24]]. These investigations frequently elucidate disparities in women's selection of menstrual materials, predicated on geographic distribution, biodemographic attributes, socioeconomic standing, and attitudes, knowledge, and practices concerning hygienic material usage among adolescent girls. Additionally, numerous studies have delved into the correlation between suboptimal menstrual hygiene management and resultant adverse health outcomes [[25], [26], [27], [28], [29]].

However, most of the previous studies in India focused on specific geographical areas, meaning the findings cannot be applied to the entire country [[30], [31], [32], [33]]. As far as we know, this study differs from previous studies as most previous studies in India have focused on rural areas, married women, and aged 15–24 years. Thus, the present study aims to discuss exclusively the factors influencing the exclusive use of MHM during menstruation among adolescent girls in urban India. Given the population-based nature of this study, its outcomes can guide public policies to improve access to menstrual hygiene products.

## 2 Database and methodology

### 2.1 Data source

The study utilized secondary data from the fifth round of NFHS-5, which was conducted between 2019-2021. The survey, conducted by the International Institute of Population Science in Mumbai, is a comprehensive sample survey covering 28 states, 8 union territories, and 707 districts across India. It is conducted periodically to gather essential data on various aspects of population, maternal and child health, and family planning. To collect the data, multistage sampling procedures were employed at the national, state/union territory, and district levels. The districts were divided into rural and urban strata. A total of 636,699 households were surveyed, and interviews were conducted with 724,115 women between the ages of 15 and 49. The survey aimed to collect information on health, socio-economic status, demographics, and other pertinent factors.

**Fig. 1:**
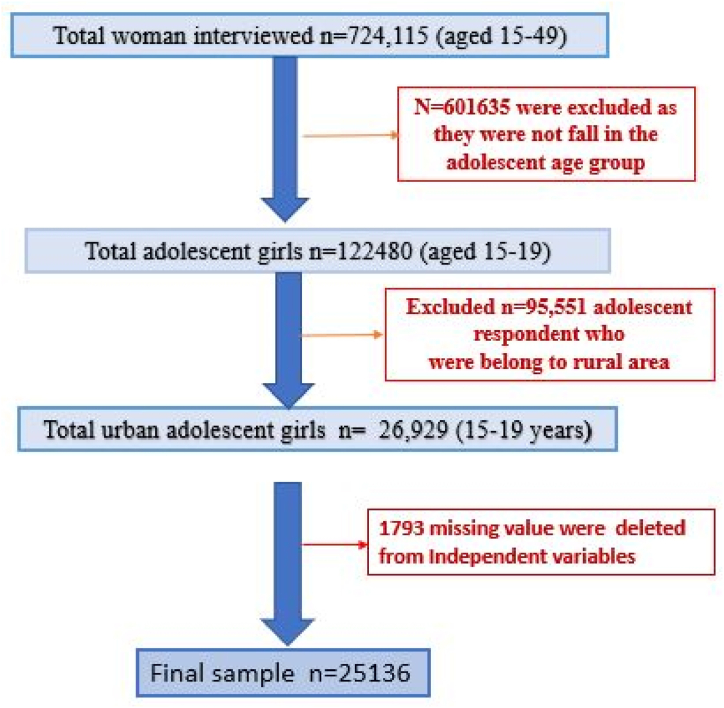
Sample selection procedure.

The NFHS-5 survey specifically collected data on menstrual practices from women aged 15 to 24. The datasets included information on menstrual protection practices, menstrual bathing practices, and socio-economic and demographic factors among women in the 15–24 age group. For the purpose of this study, urban adolescent girls aged 15–19 were selected, and the individual file of NFHS-5 provided data for 25,136 urban adolescent girls. The procedure of sample selection is presented in Fig. 1.

### 2.2 Dependent variable

The focus of our study was the exclusive use of MHM during menstruation among adolescent girls aged 15–19. In the NFHS-5 survey, adolescent girls were asked a series of multiple-choice questions to gather information about the materials they use during menstruation. Specifically, the survey question asked, "What do you use for protection?" The responses included seven options: (1) locally prepared napkins, (2) sanitary napkins, (3) tampons, (4) menstrual cups, (5) cloth, (6) nothing, and (7) others. For our analysis, we considered the usage of sanitary napkins, locally prepared napkins, menstrual cups, and tampons as hygienic products. The remaining options were categorized as non-hygienic products. Adolescent girls who utilized materials classified as '5–7′ or a combination of materials from both categories were classified as "non-exclusive use of MHM" and assigned a code of '0'. Conversely, adolescent girls who exclusively used materials from the '1–4′ category were categorized as "exclusive use of MHM" and assigned a code of '1'.

### 2.3 Independent variables

The selection of variables in this study has been meticulously curated based on an extensive review of existing literature [2,16,34,35]. These variables have been chosen as potential influencers on the exclusive use of MHM among urban adolescent girls in India. They encompass a range of vital aspects, including the age at which they experienced their first menstrual period, their educational background, religious affiliation, social group identity, household wealth, marital status, attendance at cinema or theatre, access to toilet facilities, exposure to mass media, and regional categorization. Each of these variables is pivotal in shaping the MHM practices and behaviors of the study's participants.

The respondent's age in the first period (Less than 13 and more than 13) provides valuable insights into their level of preparedness and knowledge regarding MHM. Education (No education, Primary, Secondary, and Higher) emerges as a key determinant, influencing their awareness and comprehension of MHM practices. Religion (Hindu, Muslim, Christian, and Others) and social group affiliation (General, Schedule Tribe, Schedule Caste, and Other Backward Class) are recognized as influential factors, contributing to the establishment of cultural norms and practices linked to menstruation. Household wealth (Poorest, Poorer, Middle, Richer, and Richest) assumes a critical role in determining access to resources and facilities for MHM. Marital status (Unmarried, and Married) is essential in delineating the support systems and societal norms surrounding these practices. Attendance at cinema or theatre events (Yes and No), along with mass media exposure (Yes and No), stands out as potent tools that can shape MHM knowledge. Furthermore, the presence of appropriate toilet facilities (Flush, Pit dry, Others, and No facility) emerges as an integral component of safe and dignified MHM practices. The regional categorization (North, Central, East, West, Southern, and North-east) acknowledges the nuanced variations across different regions, encompassing cultural dynamics, resource availability, and socio-economic factors, all of which contribute to the comprehensive understanding of MHM practices among urban adolescent girls in India. These variables together form a robust framework, offering deep insights into the multifaceted determinants of exclusive MHM use within this specific demographic.

### 2.4 Statistical analysis

Firstly, we conducted a descriptive analysis of the study population according to predefined variables, utilizing the chi-square test to assess differences between groups. Subsequently, to evaluate the factors associated with the use of MHM, we employed a multivariate logistic regression model, estimating odds ratios with corresponding 95 % confidence intervals. Lastly, we ensured the representativeness of the sample by applying sampling weights, and all statistical analyses were performed using STATA version 16. The model can be specified as

$$\mathrm{Log}=\left(\frac{\pi }{1-\pi }\right)=\beta_{0}+\beta_{1X_{1}}+\beta_{2X_{2}}+\ldots ∙\beta_{nX_{n}}$$

Where,

π represents the probability of an event (exclusive use of MHM),

$\beta_{0}$ represents the y-intercept,

$\beta_{1}$ represents the regression coefficients associated with the reference group, and

$x_{1}$ represents the independent variable

Odds ratios (OR) were calculated to interpret the results of the predictor variables for each outcome variable. An odds ratio greater than 1 indicates a higher probability of the outcome, while an odds ratio less than 1 indicates a lower probability. Additionally, we provided the 95 % confidence interval (CI) for the odds ratio to determine the precision of the estimates. We also assessed multicollinearity using the Variance Inflation Factor (VIF), and our study demonstrated a VIF value of 2.37, indicating the absence of multicollinearity.

## 3 Results

### 3.1 Socio-economic characteristics of respondents in the study

The study included 25,496 adolescents who practiced MHM. Among them, 20.08 % experienced the first period before the age of 13, and 79.92 % experienced the first period after the age of 13. Around 83.4 % of adolescent girls had secondary education, whereas 2.17 % had no education. Approximately 46.16 % were classified as OBC, where 27.78 %, 21.70 %, and 4.35 % belonged to general, SC, and ST communities respectively. About 75.31 % of adolescent girls identified as Hindu, while 2.40 % belonged to other religious groups and 92.74 % were unmarried adolescent girls. The respondents who belonged to the richest family were 15.29 % and 22.91 % belonged to the poorest family. The number of adolescent girls who reported attending regular cinema or theater was 17.49 %. The majority of adolescent girls used flush toilets, which was 84.80 %. Around half of the respondents frequently accessed mass media. Most of the respondents were from the Southern region, followed by the Central, west, East, Northern, and North-eastern regions, as presented in Table 1.

**Table 1:** Respondents’ characteristics and prevalence of exclusive use of MHM by socioeconomic and demographic characteristics among adolescent girls in urban India.

Background	Frequency(n)	Percentage (%)	Exclusive use of MHM				
			Frequency(n)	Prevalence	95%CI		p-value
					Lower	Upper	
**Age of first period**							0.002
Less than 13	5047	20.08	3599	71.31	69.28	73.25	
More than 13	20,089	79.92	13,735	68.37	67.04	69.67	
**Education**							<0.001
No education	546	2.17	195	35.60	29.70	41.98	
Primary	887	3.53	321	36.23	31.60	41.13	
Secondary	20,980	83.47	14,657	69.86	68.61	71.08	
Higher	2722	10.83	2161	79.39	77.07	81.53	
**Social group**							<0.001
SC	5455	21.70	3695	67.74	65.49	69.90	
ST	1094	4.35	704	64.32	59.97	6845	
OBC	11,603	46.16	7613	65.61	63.98	67.21	
General	6983	27.78	5322	76.21	74.07	78.22	
**Religion**							<0.001
Hindu	18929	75.31	13531	71.48	70.19	72.74	
Muslim	4964	19.75	2782	56.06	53.16	58.91	
Christian	641	2.55	499	77.80	72.75	82.15	
Others	602	2.40	521	86.61	83.32	89.34	
**Household wealth**							<0.001
Poorest	5758	22.91	2757	47.88	45.52	50.25	
Poorer	5702	22.69	3743	65.63	63.55	67.66	
Middle	5085	20.23	3704	72.84	70.88	74.71	
Richer	4747	18.89	3740	78.78	76.81	80.63	
Richest	3844	15.29	3391	88.22	86.64	89.63	
**Marital status**							<0.001
Unmarried	23,310	92.74	16,219	69.58	68.36	70.77	
Married	1826	7.26	1115	61.05	57.54	64.45	
**Go to cinema**							<0.001
No	20,741	82.51	13,767	66.38	65.07	67.65	
Yes	4395	17.49	3567	81.16	78.91	83.22	
**Toilet facilities**							<0.001
Flush	21,316	84.80	15,308	71.81	70.62	72.98	
Pit dry	1143	4.55	662	57.90	52.21	63.39	
No facility	1595	6.35	761	47.69	42.77	52.66	
Others	1081	4.30	603	55.82	50.86	60.65	
**Mass media exposure**							<0.001
No	11,009	43.80	6842	62.15	60.45	63.82	
Yes	14,127	56.20	10,492	74.27	72.89	75.61	
**Region**							<0.001
North	4118	16	3313	80.46	78.85	81.97	
Central	5982	24	3021	50.50	48.16	52.84	
East	4236	17	2634	62.20	58.75	65.52	
West	4366	17	3327	76.22	73.10	79.08	
Southern	6010	24	4782	79.58	77.62	81.41	
North-east	425	2	256	60.17	56.30	63.90	

CI = confidence interval, p-values are derived from Pearson's chi-square test.

**Fig. 2:**
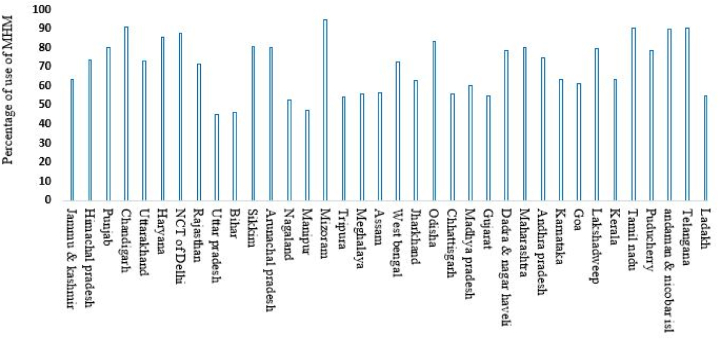
State wise reginal variation in exclusive use of MHM among adolescent girls in urban India.

Fig. 2 provides a comprehensive overview of the regional distribution of exclusive MHM practices among urban adolescent girls across various states and territories in India. In regions categorized as "Highest," where more than 78 % of adolescent girls practice exclusive MHM, states such as the National Capital Territory (NCT) of Delhi, Haryana, Chandigarh, Punjab, and Rajasthan are prominent. Conversely, the "Lowest" category includes regions where less than 62 % of adolescent girls practice exclusive MHM. States like Ladakh, Assam, Meghalaya, Tripura, Manipur, Nagaland, Mizoram, Arunachal Pradesh, and Sikkim are listed in this category, indicating significant challenges in promoting proper menstrual hygiene. In the "Medium" category, encompassing regions with MHM use ranging from 62 % to 78 %, states like Uttarakhand, Uttar Pradesh, Madhya Pradesh, Chhattisgarh, Jharkhand, Odisha, West Bengal, Bihar, Telangana, Karnataka, and Andhra Pradesh are identified.

### 3.2 Prevalence of exclusive use of MHM by different characteristics of adolescent girls (15–19 years) in urban India

Table 1 presents the prevalence of MHM across socio-demographic factors. The prevalence of these practices was slightly higher among respondents who experienced their first menstrual period before the age of 13 (71.31 %) compared to those who experienced it after the age of 13 (68.37 %). A linear relationship was observed between MHM and education levels. The percentage of respondents practicing MHM increased progressively from illiterate individuals (35.60 %) to those with higher education (79.39 %). The prevalence of exclusive MHM was significantly lower among Muslim respondents (56.06 %) in comparison to Hindu (71.48 %), Christian (77.80 %), and other religious groups (86.61 %). Among different social groups, the lowest percentage of exclusive use of MHM (64.32 %) was found among ST adolescent girls, in comparison, the highest percentage (76.21 %) was observed among adolescent girls from the general. Higher household wealth corresponds to a higher prevalence of MHM use. The prevalence of using MHM varied noticeably between respondents who attended cinema (81.16 %) and those who did not (66.38 %). Adolescent girls who had access to flush toilets (71.81 %) exhibited a higher prevalence of menstrual hygiene practices compared to those with pit-dry facilities (57.90 %), other types of facilities (55.82 %), or no facilities at all (47.69 %). Furthermore, adolescent girls with greater exposure to mass media (74.27 %) were more likely to use of MHM during menstruation. Lastly, significant regional variations in the prevalence of the use of MHM highlighted differences across different regions.

### 3.3 Socio-economic and demographic factors on exclusive use of MHM among adolescent girls (15–19 years) in urban India

Table 2 presents the results of the multivariate logistic regression analysis, which examined the impact of socio-demographic factors on exclusive use of MHM. Adolescents with a higher level of education (AOR: 2.110; 95 % CI: 1.553–2.867) and secondary education (AOR: 1.860; 95 % CI: 1.418–2.439) are more likely to use MHM exclusively. Adolescent girls belonging to the Muslim religion had a lower probability of using MHM than those of the Hindu religion (AOR: 0.576; 95 % CI: 0.520–0.637), while those of other religions had a higher likelihood (AOR: 1.509; 95 % CI: 1.164–1.957). The odds ratio was significantly higher among adolescent girls of the general (AOR: 1.255; 95 % CI: 1.103–1.429) compared to those of the SC, whereas OBC adolescent girls had a lower odds ratio (AOR: 0.858; 95 % CI: 0.771–0.955). Wealth was significantly associated with the use of MHM. Compared to the poorest adolescent girls, the odd ratio was higher in rest four category i.e. richest adolescent girls (AOR: 5.310; 95 % CI: 4.494–6.275), richer adolescent girls (AOR: 3.018.310; 95 % CI: 2.624–3.471), middle adolescent girls (AOR: 2.345; 95 % CI: 2.065–2.662), and poorer adolescent girls (AOR: 1.717; 95 % CI: 1.528–1.930). Adolescent girls who frequently attended cinema or theater had a significantly higher odds ratio (AOR: 1.338; 95 % CI: 1.181–1.517). Adolescent girls who reported no access to toilet facilities were less likely to use MHM (AOR: 0.742; 95 % CI: 0.628–0.877). The probability of using MHM was higher among adolescent girls exposed to mass media less than once a week (AOR: 1.097; 95 % CI: 1.009–1.194). As compared to the Northern region the higher odds ratio is found in the Southern region (AOR: 1.255; 95 % CI: 1.101–1.431) and the significantly lower odd ratio is found in the Western region (AOR: 0.879; 95 % CI: 0.759–1.019) Eastern region (AOR: 0.747; 95 % CI: 0.654–0.854) Central region (AOR: 0.349; 95 % CI: 0.313–0.388) and North-eastern region (AOR: 0.597; 95 % CI: 0.490–0.727).

**Table 2:** Logistic regression analysis showing the factors associated with MHM among adolescent girls in urban India.

Independent Variables	UOR	95 % CI	AOR	95 % CI
		Lower-Upper		Lower-Upper
**Age of first period**				
Less than 13®				
More than 13	0.870^a^	(0.792- 0.955)	1.01	(0.911–1.119)
**Education**				
No education®				
Primary	1.028	(0.750–1.407)	1.003	(0.723–1.391)
Secondary	4.193^a^	(3.249–5.410)	1.860^a^	(1.418–2.439)
Higher	6.967^a^	(5.248–9.250)	2.110^a^	(1.553–2.867)
**Religion**				
Hindu®				
Muslim	0.509^a^	(0.465–0.557)	0.576^a^	(0.520–0.637)
Christian	1.398^b^	(1.075–1.818)	0.971	(0.713–1.324)
Others	2.581^a^	(2.020–3.297)	1.509^a^	(1.164–1.957)
**Social group**				
SC®				
ST	0.859^c^	(0.723–1.020)	0.988	(0.806–1.212)
OBC	0.909^b^	(0.827–0.999)	0.858^a^	(0.771–0.955)
General	1.526^a^	(1.361–1.710)	1.255^a^	(1.103–1.429)
**Household wealth**				
Poorest®				
Poorer	2.079^a^	(1.871–2.309)	1.717^a^	(1.528–1.930)
Middle	2.919^a^	(2.610–3.263)	2.345^a^	(2.065–2.662)
Richer	4.040^a^	(3.582–4.558)	3.018^a^	(2.624–3.471)
Richest	8.147^a^	(7.053–9.410)	5.310^a^	(4.494–6.275)
**Marital status**				
Unmarried®				
Married	0.685^a^	(0.595–0.789)	0.938	(0.802–1.097)
**Go to cinema**				
No®				
Yes	2.182^a^	(1.946–2.446)	1.338^a^	(1.181–1.517)
**Type of toilet**				
Flush®				
Pit dry	0.540^a^	(0.457- 0.639)	0.801^b^	(0.671–0.955)
No facility	0.358^a^	(0.307–0.417)	0.742^a^	(0.628–0.877)
Others	0.496^a^	(0.414–0.594)	0.844^c^	(0.692–1.030)
**Mass media**				
No®				
Yes	1.758^a^	(1.632–1.895)	1.097^b^	(1.009–1.194)
**Region**				
North®				
Central	0.248^a^	(0.225–0.273)	0.349^a^	(0.313–0.388)
East	0.400^a^	(0.354–0.450)	0.747^a^	(0.654–0.854)
West	0.778^a^	(0.678–0.893)	0.879^c^	(0.759–1.019)
Southern	0.946	(0.844–1.061)	1.255^a^	(1.101–1.431)
North-east	0.367^a^	(0.315–0.428)	0.597^a^	(0.490–0.727)

UOR = unadjusted odds ratio, AOR = adjusted odds ratio, CI = confidence interval, ® = reference category.

a If p < 0.01.

b If p < 0.05.

c If p < 0.1.

## 4 Discussion

The findings underscore the need for targeted interventions for disadvantaged urban adolescent girls, necessitating context-specific programs and policies [36]. The study found that higher education levels, wealth quintiles, and frequent visits to cinemas were associated with increased menstrual hygiene product usage. Conversely, lack of sanitation facilities, religious groups, and social groups were linked to lower usage rates. Regional disparities were evident, with some areas exhibiting lower likelihoods of product usage. NFHS-5 (2019–2021) data indicated an increase in hygienic method usage among adolescent girls from 58 % in NFHS-4 (2015–2016) to 77 %. Despite this improvement, a significant proportion of adolescent girls and women still resort to unhygienic methods, highlighting the ongoing challenge of menstrual hygiene management.

The study findings indicate variations in the utilization of MHM among different social groups of adolescent girls in urban India. Specifically, it reveals that adolescent girls belonging to the OBC exhibit lower usage compared to those from the SC group, while adolescent girls from the general demonstrate higher usage than SC adolescent girls. Numerous studies have explored the relationship between social group identity and MHM in India [37,38]. Although findings differ across studies, some research suggests that marginalized groups, such as SC or OBC, may encounter additional obstacles in adopting appropriate menstrual hygiene practices when compared to girls from higher social castes or general. These obstacles may include limited access to hygienic products, inadequate awareness, and societal stigma associated with menstruation.

Religion significantly influences the cultural practices and beliefs related to menstruation in various societies, including India [39]. Our study revealed that the use of MHM differs among adolescent girls of different religious backgrounds in urban India. Specifically, Muslim adolescent girls demonstrated lower usage, while Hindus and girls from other religious backgrounds showed higher usage of such methods. This finding is consistent with a study, which explored cultural beliefs and practices surrounding menstruation among Hindu and Muslim adolescent girls [40]. The observed disparity in MHM utilization arises from the lower levels of knowledge and reduced likelihood of MHM adoption among Muslim girls, as contrasted with Hindu and Jain counterparts. Addressing the disparities in MHM across religious groups requires a comprehensive approach. It involves raising awareness about menstrual health, ensuring access to affordable and sustainable hygienic products, improving water and sanitation infrastructure, and challenging cultural norms and taboos associated with menstruation. By implementing these measures, promoting menstrual hygiene can be achieved, benefiting girls of all religious backgrounds.

The role of household wealth in influencing the use of MHM among adolescent girls is an important aspect to consider [41]. The level of household wealth can impact the availability of resources and affordability of hygienic materials, which in turn can affect the adoption and consistent practice of menstrual hygiene. The findings from the present study indicate that the usage of MHM varies significantly among different wealth groups of adolescent girls in urban India. Specifically, the highest usage of MHM was observed among adolescent girls in the richest quintile, followed by those in the rich, medium, poorer, and poor quintiles. Supporting evidence for this can be found in a study conducted by Sing and Anand [42], which investigated the relationship between socio-economic status and MHM among adolescent girls. The study revealed that girls from higher socio-economic backgrounds were more likely to use sanitary pads and adopt hygienic products compared to those from lower socio-economic backgrounds. Conversely, girls from lower socio-economic backgrounds often resorted to using unhygienic alternatives such as old cloth, ashes, or husk. These findings emphasize the significance of addressing socio-economic disparities and ensuring access to affordable menstrual hygiene products for adolescent girls from economically disadvantaged households [43].

Numerous studies have provided evidence supporting a positive link between higher levels of education and improved menstrual hygiene practices, which aligns with the findings of our study [44]. For instance, Anand et al. conducted a study in India and discovered a significant association between higher educational attainment among adolescent girls and better menstrual hygiene practices. It is essential to recognize that a range of factors, such as cultural norms, availability of hygiene products, socio-economic status, and awareness initiatives, can influence the connection between education and MHM.

The study uncovered another significant revelation, illustrating a notable positive correlation between frequent cinema screenings and the adoption of MHM among adolescent girls in urban India. This finding echoes and extends upon prior research, which has consistently highlighted the influential role of media exposure on menstrual hygiene behaviors [16]. Adolescent girls who attend cinema screenings may find themselves immersed in a milieu where various media platforms, including advertisements, public service announcements, or cinematic depictions, actively advocate for menstrual hygiene practices [20]. Comparatively, these findings resonate with previous studies, suggesting that exposure to diverse media channels can significantly impact attitudes and behaviors related to menstrual hygiene management among adolescent girls [25]. In corroborating existing literature, our study adds depth to the understanding of how media exposure, particularly through cinematic experiences, can catalyze fostering positive menstrual hygiene practices among adolescent girls in urban settings. This underscores the importance of leveraging media platforms as powerful tools for disseminating menstrual health education and promoting hygienic products, ultimately contributing to the overall well-being of adolescent girls in urban India. Furthermore, the study highlighted that adolescent girls who engage with mass media, such as television or radio, daily are more likely to adopt hygienic methods during their menstrual periods compared to those who do not use mass media at all. Regular exposure to mass media can play a role in increasing awareness and knowledge about menstrual hygiene practices, thus influencing the behavior and choices of adolescent girls. Consistent findings have been reported in various previous studies [[45], [46], [47]].

Our study revealed that adolescent girls in the southern region of urban India exhibit a higher prevalence of using MHM compared to girls in the Northern, Eastern, Central, Western, and Northeastern regions. This aligns with the findings of a study conducted by Dasgupta et al., which focused on West Bengal, a state in eastern India [48]. The study highlighted differences in menstrual hygiene practices among adolescent girls within West Bengal, including sanitary pads, availability of private spaces for changing menstrual materials, and access to clean water. It is essential to acknowledge that various factors, such as cultural norms, socioeconomic conditions, access to education and healthcare, and awareness about MHM can influence regional disparities in menstrual hygiene practices.

The findings of this study hold significant implications for public policies aimed at addressing the lack of access to MHM among urban adolescents, particularly those facing greater social vulnerability. Targeted interventions for marginalized groups are essential, requiring policies that prioritize subsidized or free distribution of menstrual hygiene products, educational programs, and access to proper sanitation facilities. Equitable access to affordable and quality hygiene products is crucial, requiring government subsidies or community-based distribution networks. Public policies should focus on promoting awareness and education about menstrual hygiene among urban adolescents through comprehensive school-based programs, community outreach initiatives, and media campaigns. Integration of menstrual hygiene education and services into existing health and education programs targeting adolescents can help normalize discussions about menstruation and reduce stigma. Furthermore, infrastructure development should prioritize access to clean and safe sanitation facilities in urban areas, including gender-sensitive toilets equipped for menstrual hygiene management [49]. Robust monitoring and evaluation mechanisms should be incorporated into policies to assess effectiveness and address gaps in menstrual hygiene access among urban adolescents. Additionally, policies must recognize and address intersectional inequalities, considering factors such as gender, socioeconomic status, disability, and ethnicity to ensure inclusivity and sensitivity to diverse needs [50]. Efforts from governmental initiatives, non-governmental organizations, and community-based interventions have addressed these disparities and promoted improved menstrual hygiene practices throughout India [51].

## 5 Strengths and limitations of the study

The present study exhibits several notable strengths. Firstly, using data derived from the NFHS-5 contributes to a nationally representative sample. This quality ensures the generalizability of the findings to the entirety of the urban adolescent girls in India. Secondly, the study's comprehensive examination of various factors, including education, religion, socioeconomic status, and mass media exposure, provides a holistic perspective on the determinants of menstrual hygiene practices among adolescent girls. This multi-faceted approach enhances the depth of the analysis and enriches the study's insights. Thirdly, by identifying regions within India where menstrual hygiene practices are more prevalent, the study provides crucial information for public health policymakers and managers, enabling targeted interventions and policy adjustments based on urgent needs.

However, the study is not without its limitations. Firstly, it relies on cross-sectional data obtained from NFHS-5. As a result, the study offers a snapshot in time, limiting its ability to establish causal relationships. Secondly, This study used only those variables available in the datasets. Thirdly, the use of Odds Ratios warrants consideration, as it may lead to an overestimation of results and requires cautious interpretation. Lastly, the data collection hinges on self-reporting by the study participants. This approach may introduce response bias.

## 6 Conclusion

In conclusion, this study offers a nuanced perspective on the complex landscape of menstrual hygiene practices among adolescent girls in urban India. The recognition of geographical disparities in the use of menstrual hygienic methods serves as a vital starting point for addressing this critical public health concern. It is evident that a one-size-fits-all approach is inadequate to meet the diverse needs and challenges faced by adolescent girls across different regions of the country. The multifaceted factors at play, including socio-economic, educational, and media-related aspects, call for holistic strategies. This study's findings serve as an invaluable resource for policymakers, healthcare professionals, and educators. By addressing the socio-economic inequalities, promoting education and media exposure, and considering regional nuances, it is possible to create tailored interventions and programs that can empower adolescent girls to make informed choices about their menstrual health.

## Availability of data and material

The NFHS-5 data was utilized for this research. This data is publicly available. Anyone can utilize this data without any restrictions. The link is bellow:

ttps://dhsprogram.com/methodology/survey/survey-display-541.cfm.

## CRediT authorship contribution statement

**Doli Roy:** Writing – review & editing, Writing – original draft, Visualization, Methodology, Formal analysis, Data curation, Conceptualization. **Nuruzzaman Kasemi:** Writing – review & editing, Supervision, Methodology, Formal analysis. **Manik Halder:** Writing – review & editing, Visualization, Validation, Software, Methodology, Formal analysis, Data curation, Conceptualization. **Malasree Majumder:** Writing – review & editing, Visualization, Supervision, Formal analysis.

## Declaration of competing interest

The authors declare no conflict of interest.
